# Rapid improvements in MADRS with zuranolone in major depressive disorder and postpartum depression: results from the LANDSCAPE/NEST clinical development programmes

**DOI:** 10.1192/j.eurpsy.2023.276

**Published:** 2023-07-19

**Authors:** A. H. Clayton, K. M. Deligiannidis, J. A. Ramos-Quiroga, R. Lasser, A. J. Sankoh, B. Leclair, M. Kotecha, J. Doherty

**Affiliations:** 1 Department of Psychiatry and Neurobehavioral Sciences, University of Virginia School of Medicine, Charlottesville, VA; 2 Department of Psychiatry, Zucker Hillside Hospital, Glen Oaks, NY, USA, and Feinstein Institutes for Medical Research, Northwell Health, Manhasset, NY, United States; 3 Department of Psychiatry, Hospital Universitari Vall d’Hebron, Barcelona, Spain; 4 Sage Therapeutics, Inc.; 5 Biogen, Inc., Cambridge, United States

## Abstract

**Introduction:**

Rapid-acting therapies remain an unmet need in the treatment of major depressive disorder (MDD) and postpartum depression (PPD). Zuranolone (ZRN) is being evaluated as a once-daily, oral, 14-day treatment for adult patients with MDD and PPD.

**Objectives:**

To evaluate the efficacy (assessed by Montgomery–Åsberg Depression Rating Scale [MADRS]) and safety of ZRN versus placebo across clinical studies with MDD and PPD.

**Methods:**

In 5 completed Phase 2/3 placebo-controlled randomised studies of once-daily ZRN 30 or 50 mg in adults with MDD or PPD, improvement in depressive symptoms was assessed at Day 15 (end of 14-day treatment) by change from baseline in MADRS total score and the percentage of patients achieving MADRS response (≥ 50% improvement from baseline in total score) and remission (total score ≤ 10). Safety was assessed throughout.

**Results:**

Patients in the ZRN arm achieved improvements in depressive symptoms, as assessed by MADRS. Improvements in MADRS total score at Day 15 were observed in all 5 studies and were nominally significant (p < 0.05) versus placebo in 4 studies (**Fig. 1**). Percentage of patients achieving response and/or remission in the ZRN arm was numerically greater than placebo in all MDD studies and significantly greater than placebo in the PPD studies (**Fig. 2 and 3**). ZRN was generally well tolerated with consistent safety and tolerability profiles across studies. The most common treatment-emergent adverse events (≥5 % in ZRN treatment arm) were headache, somnolence, dizziness, nausea, sedation, diarrhea, upper respiratory tract infection, fatigue, and COVID-19.

**Image:**

**Figure:**
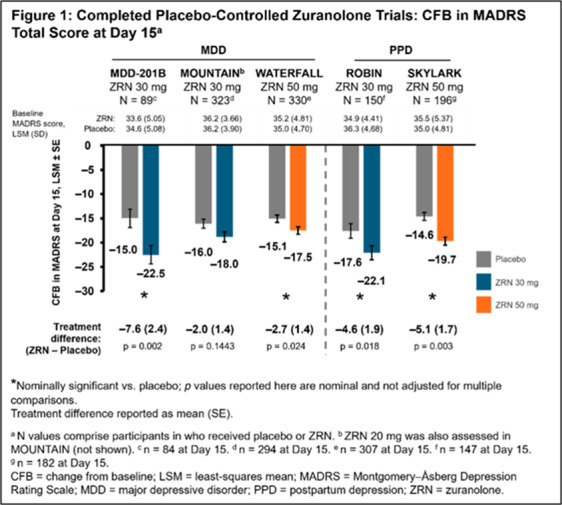

**Image 2:**

**Figure:**
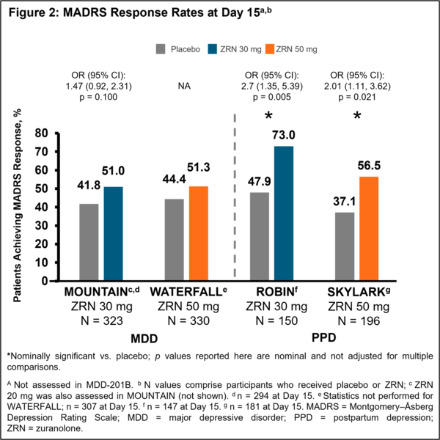

**Image 3:**

**Figure:**
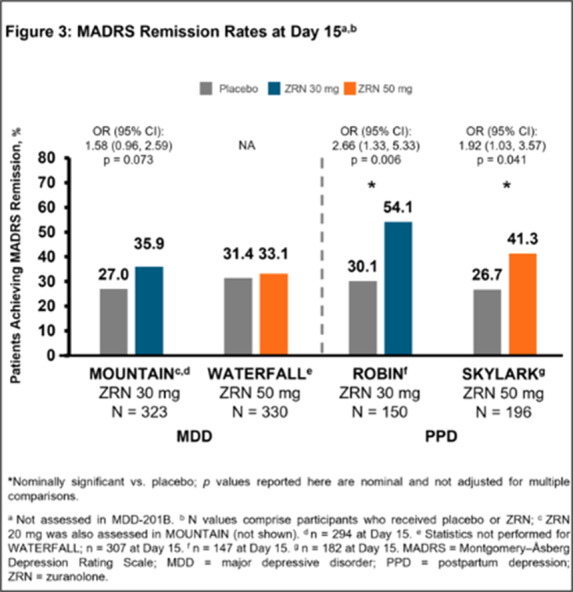

**Conclusions:**

In the 5 completed clinical studies, rapid improvement in depressive symptoms assessed by MADRS was observed across studies of adults with MDD and PPD who received a 14-day treatment of once-daily ZRN. In all studies, ZRN was generally well tolerated. These data support further development of ZRN as a potential oral, rapid-acting treatment for patients with MDD or PPD.

**Funding:**

The MDD-201B, MOUNTAIN, and ROBIN studies were sponsored by Sage Therapeutics, Inc.; the WATERFALL and SKYLARK studies were sponsored by Sage Therapeutics, Inc. and Biogen Inc. Medical writing and editorial support were provided by MediTech Media, Ltd, and funded by Sage Therapeutics, Inc. and Biogen Inc.

**Disclosure of Interest:**

A. Clayton Shareolder of: Royalties from Ballantine Books/Random House, the Changes in Sexual Functioning Questionnaire, and Guilford Publications; and restricted stock in Euthymics, Mediflix LLC, and S1 Biopharma., Grant / Research support from: Daré Bioscience, Janssen, Otsuka, Praxis Precision Medicines, Relmada Therapeutics, Inc., and Sage Therapeutics, Inc, Consultant of: AbbVie, Inc., Brii Biosciences, Inc., Fabre-Kramer, Janssen Research & Development, LLC, Mind Cure Health, Ovoca Bio plc, Praxis Precision Medicines, PureTech Health, Reunion Neuroscience (formerly Field Trip Health) S1 Biopharma, Sage Therapeutics, Inc., Takeda/Lundbeck, Vella Bioscience, Inc., and WCG MedAvante-ProPhase, K. Deligiannidis Shareolder of: Royalties from an NIH employee invention outside of the submitted work. , Grant / Research support from: Received grants from from NIH and Vorso Corporation. Grants awarded to Zucker Hillside Hospital/Feinstein Institutes for Medical Research during the conduct of the brexanolone injection and zuranolone clinical trials (Sage Therapeutics), Consultant of: Sage Therapeutics, Inc., Brii Biosciences, Inc., and GH Research Ireland Limited, J. A. Ramos-Quiroga Grant / Research support from: The Department of Mental Health chaired by him received unrestricted educational and research support from the following companies in the last 3 years: Janssen-Cilag, Shire, Oryzon, Roche, Psious, and Rubió. Dr Ramos-QuirogaReceived travel awards (air tickets + hotel) for taking part in psychiatric meetings from Janssen-Cilag, Rubió, Shire, Takeda, Shionogi, Bial, and Medice., Consultant of: Was on the speaker’s bureau and/or acted as consultant for Janssen-Cilag, Novartis, Shire, Takeda, Bial, Shionogi, Sincrolab, Novartis, BMS, Medice, Technofarma, Rubió and Raffo in the last 3 years., Speakers bureau of: Was on the speaker’s bureau and/or acted as consultant for Janssen-Cilag, Novartis, Shire, Takeda, Bial, Shionogi, Sincrolab, Novartis, BMS, Medice, Technofarma, Rubió and Raffo in the last 3 years., R. Lasser Shareolder of: May hold stock and/or stock options of Sage Therapeutics, Inc. , Employee of: Employee of Sage Therapeutics, Inc., A. Sankoh Shareolder of: May hold stock and/or stock options of Sage Therapeutics, Inc., Employee of: Employee of Sage Therapeutics, Inc. , B. Leclair Shareolder of: May hold stock of Biogen Inc., Employee of: Employee of Biogen Inc., M. Kotecha Shareolder of: May hold stock of Biogen Inc., Employee of: Employee of Biogen Inc., J. Doherty Shareolder of: May hold stock and/or stock options of Sage Therapeutics, Inc., Employee of: Employee of Sage Therapeutics, Inc.

